# Use of Loop-Mediated Isothermal Amplification in a Resource-Saving Strategy for Primary Malaria Screening in a Non-Endemic Setting

**DOI:** 10.4269/ajtmh.18-0496

**Published:** 2019-01-21

**Authors:** Gitte N. Hartmeyer, Silje V. Hoegh, Marianne N. Skov, Michael Kemp

**Affiliations:** 1 Research Unit of Clinical Microbiology, Department of Clinical Research, Faculty of Health Science, University of Southern Denmark, Odense, Denmark;; 2 Department of Clinical Microbiology, Odense University Hospital, Odense, Denmark

## Abstract

Malaria is traditionally diagnosed by blood smear microscopy, which requires continuous resource-demanding training. In areas with only a few cases of malaria, a simple and rapid test that can reliably exclude malaria could significantly reduce the need for microscopy and training. We evaluated whether loop-mediated isothermal amplification (LAMP) for screening malaria parasites could reduce the workload in the diagnosis of malaria. Loop-mediated isothermal amplification was used to analyze 38 ethylene-diamine-tetraacetic acid (EDTA) blood samples from 23 patients who had previously been tested for malaria by microscopy, antigen-based rapid diagnostic test (antigen-RDT), and in-house real-time polymerase chain reaction (RT-PCR). The samples included blood with low-level parasitaemia and samples with discrepancies between the results of the different methods. Loop-mediated isothermal amplification detected malaria parasites in 27 of 28 samples that were positive according to in-house RT-PCR. There were negative microscopy results in 10 of these and negative antigen-RDT results in 11. The sample with a negative LAMP result and positive in-house RT-PCR result was from a patient who had recently been treated for low-level *Plasmodium falciparum* malaria parasitaemia. We found LAMP to be reliable for malaria screening and suitable for replacing microscopy without loss of performance. The low number of LAMP-positive samples needing microscopy can be handled by a limited number of trained microscopists. The time saved on training and documentation was estimated to be 520 working hours yearly in our laboratory. Using LAMP for primary screening of patient samples, we have made a diagnostic workflow that ensures more reliable, faster, and less resource-demanding diagnosis of malaria.

## INTRODUCTION

Malaria is a serious infectious disease caused by five human pathogenic species of *Plasmodium* parasites.^1–4^ Malaria may be a life-threatening condition, especially in nonimmune individuals, and rapid and accurate diagnostic tests for malaria are essential for early and adequate treatment.^5–9^ Rapid and reliable exclusion of malaria is essential for the diagnosis of patients with relevant exposure and severe infections other than malaria.^10^

Malaria is a rare condition in Denmark, with only 101 cases in 2016 in a population of 5.7 million. Of these cases, 86% were imported from Africa and 28 percent of patients were immigrants and almost entirely from Eritrea.^11^ Traditionally, malaria is diagnosed by blood smear microscopy,^12^ which is often combined with an antigen-based rapid diagnostic test (antigen-RDT).^5,13^ However, microscopy is laborious and time consuming, and requires highly skilled staff. Sufficient microscopic examination of a blood smear takes around 60 minutes for the first sample from a patient before it can be reported as negative.^14^ Both the Centers for Disease Control and Prevention^15^ and the Clinical and Laboratory Standards Institute^16^ recommend analyzing three individual samples before excluding malaria. The sensitivity for microscopy is 20–50 parasites/μL, but it depends on the skills of the individual microscopist.^6^ When using microscopy as the only tool for detecting malaria parasites, cases with low parasitemia are easily missed.

In countries with only a few cases of malaria, much time is spent on microscopy for negative samples. Obtaining, maintaining, and documenting microscopy skills are time consuming and expensive for diagnostic laboratories in non-endemic countries. Alternatives to microscopy include antigen-RDT and real-time polymerase chain reaction (RT-PCR). Antigen-RDT is fast and easy but has lower reported sensitivity than microscopy, and the technique is, therefore, not suited for eliminating a diagnosis of malaria.^17^

The analytical sensitivity of RT-PCR is < 1 parasite/μL.^18–21^ Most PCR systems take 3–6 hours before results are available, making them unsuitable for initial screening. Quantification of parasitemia by RT-PCR is notoriously difficult.^20,22^ A simple screening test that can exclude malaria with high reliability would reduce the need for microscopy to only samples that test positive to establish the species and the number of infected erythrocytes. This would reduce the number of individuals who need to maintain skills in malaria microscopy.

Different loop-mediated isothermal amplification (LAMP) methods have recently been introduced to the market to detect malaria parasites. Loop-mediated isothermal amplification analysis takes less than 1 hour and has comparable sensitivity to RT-PCR.^6,7,23–29^ Loop-mediated isothermal amplification is a one-step molecular amplification technique with non-malaria species qualitative results. The test is very simple to perform and does not require advanced equipment. Loop-mediated isothermal amplification was introduced as a point-of-care primary diagnostic test for screening malaria in endemic settings.^30,31^ Polley et al.,^23^ Rypien et al.,^24^ Konincik et al.,^25^ Ponce et al.,^29^ and most recently Frickmann et al.^32^ showed that the use of LAMP to diagnose malaria in a non-endemic setting both improved diagnostic sensitivity and saved time. The aim of this study was to investigate whether LAMP could replace microscopy for malaria screening without loss of performance and to estimate the savings that can be obtained from a change in screening technology.

## MATERIAL AND METHODS

### Setting.

The laboratory provides diagnostic service to a population of approximately 500,000 individuals, including a university hospital with 1,000+ beds. All patients in the Region of Southern Denmark (1.2 million inhabitants) with confirmed malaria are referred to the university hospital for treatment. The standard testing for malaria consists of microscopy in combination with antigen-RDT. The annual number of positive samples in the period 2006–2017, the total number of samples analyzed for malaria parasites in 2016, and the time of day when they were requested were all extracted from the laboratory information system.

### Microscopy.

Blood was routinely examined using thick and thin smears of capillary blood stained with 4% Giemsa. The first-time microscopic examination for malaria was always carried out immediately on the arrival of the sample. During normal working hours, a second examination was carried out on all first-time samples immediately after the first examination. Samples examined outside normal working hours were reexamined in the morning on the next day. Follow-up samples were examined on the day of arrival or the following day if received at night.

### Resources used for obtaining and documenting microscopy skills.

The 60 laboratory technicians for malaria microscopy require 8 hours of training and testing. They are tested four times a year for 3 hours to document their microscopy skills as a part of the laboratory’s quality assurance program. Approximately five of the technicians are replaced each year. All medical doctors on call used by the department receive the same training and testing for mandatory qualifications in microscopy.

### Sample collection.

Between September 2014 and May 2017, 38 EDTA blood samples and corresponding blood films were tested. The samples were from 23 patients with a history of travel to areas where malaria is endemic and were referred to malaria testing on clinical grounds. Samples were selected to represent positive microscopy samples (*Plasmodium falciparum*, *Plasmodium ovale*, *Plasmodium vivax*, and *Plasmodium malariae*) and negative microscopy samples. Samples with low parasitaemia and microscopy-negative samples with positive in-house RT-PCR results and repeated samples for some patients were included (Table 1).

**Table 1:** Results obtained by microscopy, antigen-RDT, LAMP, and RT-PCR

Sample no.	Pt.	Sample	Day	Microscopy		Antigen-RDT		LAMP	RT-PCR	
				*Plasmodium*	Stage or parasitemia	Pan band	Pf band	Pos. or neg.	Pan	Species specific
1	A	1/7	0	*P. ovale*	tro + gam	**neg**	**neg**	pos	pos	*P. ovale*
2	B*	1/4	0	**neg**	tro.+ sch + gam	**neg**	**neg**	pos	pos	*P. malariae*
3		3/4	8	*P. malariae*	tro.+ sch + gam	neg	neg	pos	pos	*P. malariae*
4	C	2/6	1	*P. vivax*	tro + gam	pos	neg	pos	pos	*P. vivax*
5		6/6	14	neg		NT		neg	neg	neg
6	D†	1/4	0	**neg**		**neg**	**neg**	pos	pos	*P. falciparum*
7		3/4	1	**neg**		**neg**	**neg**	pos	pos	*P. falciparum*
8	E	1/1	0	neg		neg		neg	neg	neg
9	F	3/4	2	**neg**		**neg**	**neg**	pos	pos	*P. vivax*
10		4/4	27	neg		neg	neg	neg	neg	neg
11	G‡	2/3	6	**neg**		**neg**	**neg**	pos	pos	*P. vivax*
12		1/3	0	*P. vivax*	tro + gam	pos	neg	pos	pos	*P. vivax*
13		2/3	1	*P. vivax*	tro + gam	**neg**	**neg**	pos	pos	*P. vivax*
14		3/3	2	*P. vivax*	gam	pos	neg	pos	pos	*P. vivax*
15	H	2/4	5	*P. vivax*	tro + gam	pos	neg	pos	pos	*P. vivax*
16	I	2/3	1	*P. vivax*	gam	pos	neg	pos	pos	*P. vivax*
17	J	3/3	2	*P. ovale*	tro + gam	pos	neg	pos	pos	*P. ovale*
18	K§	2/4	1	**neg** (known Pf)		**neg**	pos	pos	pos	*P. falciparum*
19	L	1/6	0	*P. falciparum*	<1%	**neg**	pos	pos	pos	*P. falciparum*
20		2/6	1	*P. falciparum*	<1%	pos	pos	pos	pos	*P. falciparum*
21		3/6	2	*P. falciparum*	<1%	pos	pos	pos	pos	*P. falciparum*
22		4/6	4	**neg**		**neg**	pos	pos	pos	*P. falciparum*
23		5/6	5	**neg**		**neg**	pos	pos	pos	*P. falciparum*
24		6/6	12	*P. falciparum*	gam	**neg**	pos	pos	pos	*P. falciparum*
25	M	1/2	0	neg		neg	neg	neg	neg	
26	N	1/4	0	*Plasmodium* sp.	Only trof. in thick smear	**neg**	pos	pos	pos	*P. falciparum*
27		3/4	4	**neg**		**neg**	pos	pos	pos	*P. falciparum*
28	O	1/3	0	neg		neg	neg	neg	neg	
29	P‖	4/4	6	Unclear microscopy		**neg**	**pos**	neg	Weak pos (ct > 40)	*P. falciparum*
30	Q	1/4	0	neg		neg	neg	neg	neg	
31	R	1/5	0	*Plasmodium* sp.	Only trof. in thick smear	**neg**	**neg**	pos	pos	*P. malariae*
32	S	1/3	0	neg		neg	neg	neg	neg	
33	T	3/5	2	*P. falciparum*	0.1%	pos	pos	pos	pos	*P. falciparum*
34		4/5	3	*P. falciparum*	<0.01%	**neg**	pos	pos	pos	*P. falciparum*
35		5/5	7	**neg**		**neg**	**neg**	pos	pos	*P. falciparum*
36	U	1/4	0	neg		neg	neg	neg	neg	
37	V	1/3	0	neg		neg	neg	neg	neg	
38	W	2/3	1	neg		neg	neg	neg	neg	

gam = gametocytes; LAMP = loop-mediated isothermal amplification; neg. = negative; Pan band = detection for Pf, Pv, Po, and Pm; Pf = *Plasmodium falciparum*; Pf band = specific for Pf detection; Pm = *Plasmodium malariae*; Po = *Plasmodium ovale*; pos. = positive; Pv = *Plasmodium vivax*; RDT = rapid diagnostic test; RT-PCR = real-time polymerase chain reaction; sch = schitzonts; tro = trophozoites. Discordant results are marked in bold.

* Unrecognized *P. malariae* infection until day 8 where re-microscopy was found positive for *P. malariae.*

† Unrecognized *P. falciparum* infection.

‡ First event mid-August; second event early December (treated sufficiently with malarone and primaquine between the first and second events).

§ Positive in the first sample with only a few trophozoites in thick smear.

‖ Known positive patient from another laboratory in smears 1, 2, and 3 with % parasitemia results of 0.65, 0, 1, and negative, respectively.

Based on travel history and microscopy, *Plasmodium knowlesi* was not suspected in any of the samples and was not specifically tested. We have subsequently tested the LAMP system successfully using control samples with *P. knowlesi* (data not shown).

### Rapid diagnostic test.

EDTA blood samples were tested for pan-*Plasmodium* and *P. falciparum* antigens by RDT (first Response^®^ Malaria Ag, pLDH/HRP2 Combo Card Test; Premier Medical Corporation Private Limited, Kachigam, Nani Daman, India) along with Giemsa staining in parallel. One sample was not tested by antigen-RDT on admission to the laboratory.

### In-house RT-PCR assays.

DNA was extracted from EDTA blood using a DNA and Viral NA Small Volume kit with a MagNA Pure 96 extraction system (Roche Molecular Diagnostics^©^, Pleasanton, CA) following the Pathogen Universal 200 protocol and the manufacturer’s protocol. Thirty-one of the samples had been stored at −80°C for up to 3 years, and seven had been stored at 4°C for up to 14 days. All samples were tested by pan-*Plasmodium* assay using in-house RT-PCR.^19^ Positive samples were further analyzed by species-specific in-house RT-PCR assays for *P. falciparum*,^33^ *P. vivax*,^33^ *P. ovale*,^34^ and *P. malariae*.^35^

Amplifications were performed using the 7500 FAST RT-PCR system (Thermofisher Scientific, Waltham, MA). The 25-μL reaction mixture contained 1× TaqMan^®^ Fast Universal PCR Master Mix, 2× No AmpErase^®^ UNG (Thermofisher Scientific), 1,000 nM of the primers, 200 nM of the probes, and 5 μL DNA eluate.

The reactions were carried out in singleplex using the following cycling conditions: 95°C for 20 seconds followed by 45 cycles of 95°C for 3 seconds and 60°C for 30 seconds. ROX (6-carboxy-X-rhodamine) was used as a reference dye.

### Loop-mediated isothermal amplification assay.

The EDTA blood samples were tested retrospectively with illumigene Malaria^®^ (Meridian Bioscience, Cincinnati, OH) using an Illumipro-10^™^ incubator/reader (Meridian Bioscience).

The analysis was performed according to the manufacturer’s protocol as a single test per sample (http://www.meridianbioscience.eu/media/pdf/Package%20Insert/280925_281125_MULTI_REV1215.pdf).

The assay is qualitative for the direct detection of *Plasmodium* species DNA, and the results can be reported as positive, negative, or invalid. The assay targets a 214–base pair sequence of a *Plasmodium* spp. mitochondrial DNA noncoding region that is conserved across *P. falciparum*, *P. vivax*, *P. ovale*, *P. malariae*, and *P. knowlesi*. The exact sequences of the six different primers are not available from Meridian Bioscience. The total reaction volume in the test sample is 50 μL, and the loop-mediated DNA amplification is carried out with an isothermal temperature at 63°C for 40 minutes on a combined incubator and reader.

Change in reaction solution absorbance characteristics is created by precipitation of magnesium pyrophosphate and indicates the presence of target DNA.

## RESULTS

### Resource savings for obtaining and documenting microscopy skills by replacing microscopy with LAMP.

Approximately 520 hours would be spent on quality assurance of microscopy skills for technicians each year if 60 technicians continued practicing acute microscopy (60 technicians × 2 hours × four tests + five training sessions × 8 hours). When using LAMP instead of microscopy for screening, each laboratory technician would be trained for less than 2 hours. The procedure is very simple and similar to other rapid molecular tests in the laboratory, so no further testing is planned. Thus, only new staff members (approximately five per year) need 2 hours of training. This saves a total of 510 working hours per year (almost 14 weeks), which was calculated based on the 37-hour work week in Denmark.

### Need for microscopy when using LAMP for screening.

With the expectation of examining only LAMP-positive samples by microscopy, the need for microscopy was estimated from the number of positive tests in previous years. Data extracted from the laboratory information system indicated 57 positive patients from 2014 to 2017. In 2016, the laboratory examined 361 samples from 154 patients, who were admitted to the hospital on suspicion of malaria. There were 30 positive samples from 13 patients according to microscopy. Malaria testing was mainly requested during normal working hours, and only seven samples were positive between 4:00 pm and 7:00 am in 2016 and 2017.

### Detection of malaria parasites by microscopy, RDT, RT-PCR, and LAMP.

Eighteen of the 38 samples were positive according to microscopy, and 18 of 37 blood samples were positive according to antigen-RDT. Five of the samples with negative antigen-RDT results had positive microscopy results (with *Plasmodium malaria* identified in three samples and with *P. vivax* and *P. ovale* identified in one sample each). Twenty-eight of the 38 blood samples were positive according to RT-PCR, and 27 of these were positive according to LAMP. There were 10 samples with negative microscopy results that were positive according to both RT-PCR and LAMP. Discordant results from microscopy, RDT, RT-PCR, and LAMP are summarized in Table 1.

## DISCUSSION

The growing demand for faster, better, and more efficient solutions to diagnostic challenges prompted us to explore opportunities to improve the diagnosis of malaria. Malaria is a potentially deadly infection, and access to reliable testing for patients with relevant exposure at any time is required. In non-endemic countries, most samples are negative, and maintaining and documenting skills to perform microscopy for parasites impose burdens on health budgets.

Microcopy is relatively fast but has some limitations, especially in samples with low parasitaemia. Antigen-RDT is fast and easy but has lower reported sensitivity than microscopy, and the technique is, therefore, not suited for eliminating the diagnosis of malaria.^17^ Real-time polymerase chain reaction is very sensitive, but most PCR assays take 3–6 hours before results can be obtained. Loop-mediated isothermal amplification is rapid to perform, and reports on its performance make this technique an attractive method for first-line screening.

We calculated that considerable time could be saved for maintaining and documenting skills if the need for microscopy could be reduced and handled by a few individuals. Trained doctors are on call, and the extra workload is acceptable and almost entirely within normal working hours. Compared with microscopy, LAMP requires less hands-on time and shorter total turnaround time. Rypien et al.^24^ estimated that an overall change from microscopy and antigen-RDT to LAMP would reduce costs by US$13 per patient. They found that the change would reduce both the hands-on time and cost per analysis.

We compared LAMP readings with results obtained by microscopy, antigen-RDT, and in-house RT-PCR for material obtained from travelers to endemic areas. Concordant results were obtained by RT-PCR and LAMP from all samples except one, which was positive in RT-PCR (with a threshold cycle (CT) value of 40.2) and negative in LAMP. In the thick smear, only one trophozoite was observed, whereas the RDT result was positive. On retesting, the LAMP result became positive. The patient had received treatment for *P. falciparum* infection before testing.

We were able to confirm the results from Rypien et al.,^24^ Koninck et al.,^25^ and Ponce et al.,^29^ as we also found that LAMP has comparable sensitivity to RT-PCR. A recent study by Frickmann et al.^32^ found that LAMP is suitable for initially screening patients with suspected malaria in a non-endemic setting.

We conclude that LAMP is well suited as a screening tool for malaria in a non-endemic setting. The loop-mediated isothermal amplification–positive samples should subsequently be examined by blood smear microscopy to establish species and parasitaemia. Because only a minority of samples will be positive and require microscopy, a considerable amount of time will be saved. Optimal patient management requires diagnostic procedures that are fast and inexpensive, require few resources, and are reliable.

To ensure sufficient diagnostic sensitivity of microscopy for malaria parasites, it is recommended that negative results from first-time tests be followed by at least two additional separate tests.^15,16^ The reliability and high sensitivity of LAMP may allow final conclusions from the first samples without the need for follow-up. Studies are underway to clarify this.

**Figure 1.**
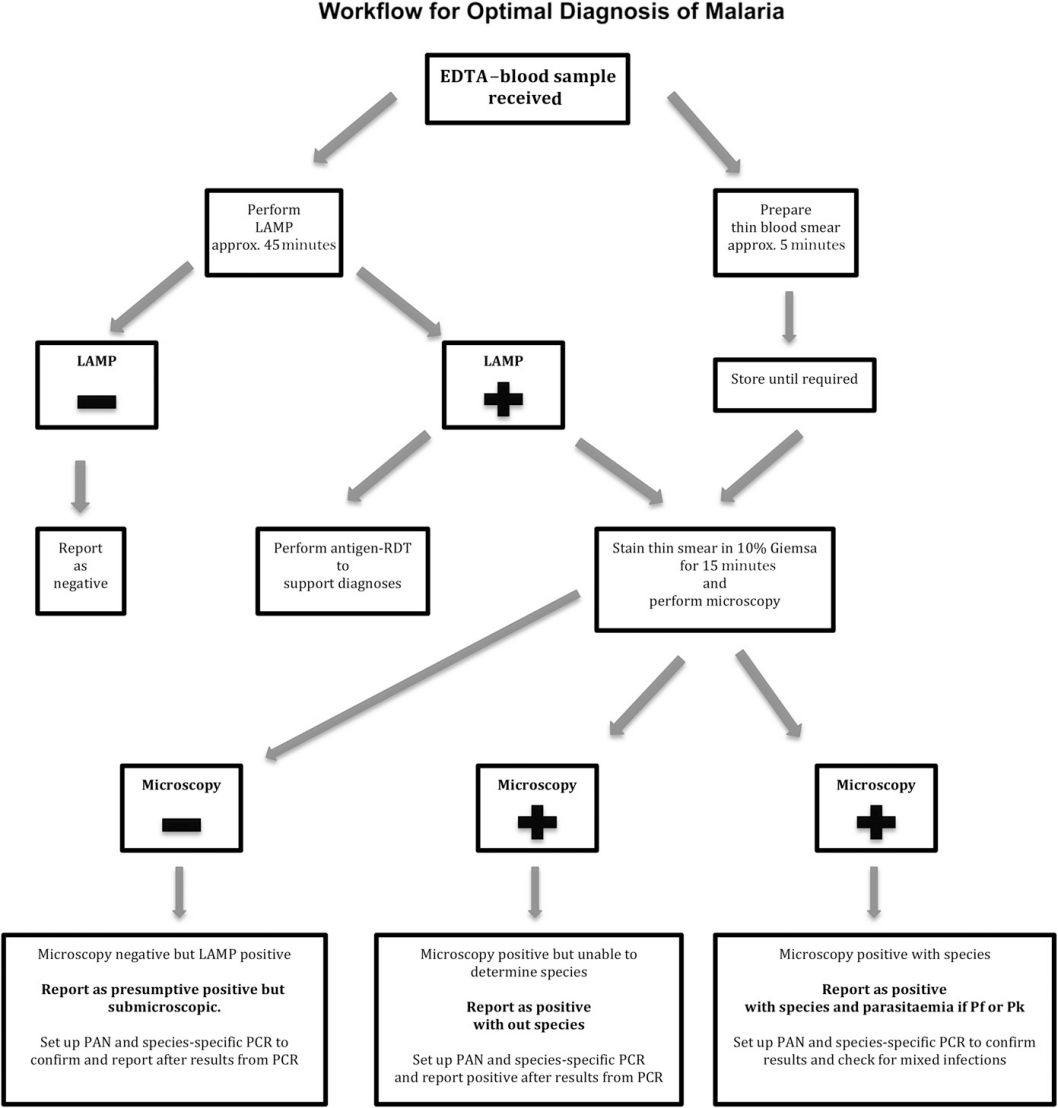
LAMP = loop-mediated isothermal amplification; PCR = polymerase chain reaction; RDT = rapid diagnostic test. Abbreviation supplemented with: PAN = in-house RT-PCR for detection of *Plasmodium* spp.; Pf = *P. falciparum; P. knowlesi*.

By using LAMP, screening for malaria can be carried out by staff without specific training in malaria microscopy. In a non-endemic setting, most samples will be negative and require no further tests. We have developed a workflow that uses LAMP for initial screening, microscopy for acute species determination and quantification, and species-specific RT-PCR for confirmation (Figure 1). This procedure ensures optimal diagnosis of malaria, requires minimal training resources, and is feasible in 24-hour 7-day laboratory services. The algorithm for malaria testing has been used by the laboratory since January 1, 2018.
